# Supplementary material for: Are carbon emissions trading and green financial instruments synergistic? -Comprehensive quantitative research based on content analysis
Source: PLoS One. 2024 Mar 7;19(3):e0298601. doi: 10.1371/journal.pone.0298601 (PMC10919601; doi:10.1371/journal.pone.0298601)
Supplement: S1 Data — (ZIP) [file pone.0298601.s001.zip › Data/地级市Z_F工作报告.zip!/╜¡╬≈/╝¬░▓╩╨/└┤╫╘╣1⁄2╓┌║┼í╛┬φ┐╦╩2╛▌═°í┐_2017.docx]

各位代表：

　　现在，我代表市人民政府向大会报告工作，请予审议，并请各位市政协委员和列席会议的同志提出意见。

　　一、2016年工作回顾

　　2016年是“十三五”开局之年，也是市县乡集中换届之年。一年来，在市委的坚强领导下，全市上下坚持以习近平总书记视察吉安、井冈山为强大动力，感恩奋进，砥砺前行，统筹做好稳增长、调结构、促改革、优生态、惠民生等各项工作，较好完成了年度目标任务，做到了“换届不松劲、工作不断档、发展不停步”，实现了“十三五”的良好开局。

　　———增长稳健。全市有13项主要经济指标增幅位居全省前三，完成生产总值1461.4亿元、增长9.2%，连续15个季度位列全省前三；财政总收入229.1亿元，同口径增长10.9%，增幅高出全省平均水平1.3个百分点。

　　———质量向好。粮食总产量415万吨，购销量居全省第一；特色富民产业加快发展，井冈蜜柚新增5.5万亩、达到30万亩。规模工业增加值760.8亿元、增长9.4%，电子信息产业主营业务收入746亿元、增长19.2%，高新技术产业增加值增长13.4%。服务业增加值占GDP比重提高1.37个百分点，三次产业结构优化为16:48.5:35.5。

　　———要素集聚。争取转移支付207亿元、用地指标2.3万亩。金融机构贷款余额1273亿元、增长20.6%。人才工作取得突破，1人入选国家“千人计划”，2人入选国家“万人计划”。

　　———活力增强。创新驱动深入实施，全市R＆D经费支出①增加5.3亿元，占GDP比重达到0.89%。专利授权量增长90.3%，增幅居全省第二。

　　———民生改善。新增城镇就业5.6万人，完成省下达任务的124.8%。城镇登记失业率2.9%，低于控制目标1.6个百分点。居民消费价格指数上涨1.3%，控制在目标范围内。城镇居民人均可支配收入增长8.2%、达到29307元，农村居民人均可支配收入增长9.9%、达到11380元。

　　一年来，我们主要做了以下工作：

　　（一）综合施策应对下行压力，促进了经济新增长。加大实体经济帮扶力度，深入开展“降成本、优环境”专项行动，全面落实省“100条”，制定市“26条”，累计为企业减负47亿元，促进了企业降本增效、转型升级。扭住项目建设不放松，一批重大产业项目落地见效，木林森二极管项目一期全面投产，井冈山卷烟厂易地技改竣工运营，百威英博一期正式投产；一批打基础、管长远的交通能源项目加快推进，新井冈山大桥竣工通车，东乡至昌傅高速吉安段全面建成，广昌至吉安高速、井冈山航电枢纽开工建设。全市完成固定资产投资1710亿元、增长15%，特别是出台支持民间投资“39条”，民间投资占全部投资比重达到68.8%。

　　（二）坚定不移聚焦改革开放，释放了发展新活力。坚持以供给侧结构性改革作为经济工作主线，全面完成煤炭、钢铁行业去产能年度任务，出台房地产去库存“18条”，全市商品住宅库存去化时间控制在6个月以内，比2015年底减少8个月。深化政府改革，精简行政审批事项49项，取消和调整行政审批中介服务事项12项，权责清单制度进一步向乡镇延伸。推进全面开放，市本级先后在北上广深等地举办重大招商活动12场次，在深圳举办的股权招商开全省先河，全市实际利用外资9.75亿美元、增长10.4%，引进省外资金586.6亿元、增长13.1%，实现外贸出口280.3亿元、增长8.2%。

　　（三）深处着力推进创新创业，激发了发展新动能。大力实施创新驱动发展战略，新增省级以上创新平台载体12个、院士工作站和“海智计划”②工作站各1个，国家级科技孵化器实现“零突破”，全市高新技术企业达135家。启动实施“五证合一”③、“一照一码”登记制度，深入推进“大众创业、万众创新”，个体工商户和私营企业分别增长11.8%、17.4%。加强金融创新，政银企合作不断深化，进军资本市场取得历史性突破，7家企业实现“新三板”挂牌，42家企业在区域性股权交易市场挂牌，企业直接融资突破200亿元。

　　（四）统筹推进区域城乡发展，优化了发展新格局。区域格局进一步优化，井冈山经开区主营业务收入突破600亿元、在全省排名再进1位，吉安高新区主营业务收入达到350亿元、增长13.6%；吉泰走廊发展迅速，生产总值和财政总收入占全市比重分别提高0.5和0.6个百分点。城乡结构进一步优化，全市城镇化率提高1.6个百分点、达到47.8%，中心城区建成区面积新增3平方公里、达到75平方公里，公共自行车系统投入使用，创建全国文明城市迎接国检，控违拆违扎实有效，整治各类广告1.9万块；美丽乡村建设扩面提质，新增964个美丽乡村精品点，城乡面貌展现新景象。

　　（五）持续加强生态文明建设，提升了绿色新优势。深入开展“净空”行动，淘汰黄标车及老旧机动车1.2万辆、超额完成省下达任务。“河长制”④全面推行，“清河行动”成效明显，主要河流断面水质达到国家标准。耕地保护持续加强，全面实行基本农田永久保护和红线管控。节能减排力度加大，万元GDP能耗全省最低。成功获批国家循环经济示范城市、全国低碳试点城市，新增4个县纳入国家重点生态功能区，万安湖入选国家湿地公园，井冈山市、永新县获评全省第二批生态文明示范县。

　　（六）不遗余力改善民生福祉，顺应了群众新期待。民生投入持续加大，全市民生支出309.6亿元，占总支出的80.9%。脱贫攻坚首战告捷，整合投入涉农扶贫资金33亿元，派出驻村干部3万多名，全市279个贫困村退出，减少贫困人口11.3万人，井冈山市、吉安县有望在全省率先脱贫摘帽。安居工程扎实推进，棚户区改造开工1.07万套。文教卫生事业加快发展，超额完成“直播卫星户户通”任务，新增7个县区通过国家义务教育均衡发展督导评估；医疗卫生服务能力进一步提升，免费救治贫困家庭大病患者1.9万例；食品药品安全监管有效加强；全民健身活动广泛开展。平安吉安、法治吉安建设深入推进，安全生产形势总体平稳，公众安全感继续位居全省前列，社会保持和谐稳定。

　　一年来，我们着力加强政府自身建设，转职能、改作风，政府工作效率和服务水平进一步提高。扎实开展“两学一做”学习教育，严格落实中央八项规定精神和国务院“约法三章”。深入推进法治政府建设，严格依法行政，市政府常务会议专题学法8次。大力推进廉洁政府建设，整治查处了一批侵害群众利益的不正之风和腐败问题。

　　各位代表，过去一年，全市人民积极进取，奋发有为，推动各项事业取得新进步。成绩来之不易，这是省委、省政府和市委坚强领导、科学决策的结果，是全市人民攻坚克难、砥砺奋进的结果，是社会各界关心支持的结果。在此，我代表市人民政府，向在各个工作岗位上贡献聪明才智、付出辛勤汗水的全市广大干部群众，向各民主党派、工商联、无党派和社会各界人士，向驻吉人民解放军、武警官兵和公安消防干警，向所有关心支持吉安发展的同志们、朋友们，致以衷心的感谢和崇高的敬意！

　　需要向代表说明的是，受经济下行压力持续加大等因素影响，全市生产总值、规模工业增加值、外贸出口、居民收入等指标增长低于预期目标。同时，我们也清醒认识到，我市经济社会发展中还存在不少困难和问题，主要是：经济稳定运行的基础还不牢固，民间投资增长乏力，招商引资难度加大；实体经济困难不少，融资难、融资贵问题依然突出；产业基础不稳，创新能力不强；少数干部思想保守、作风漂浮，缺乏进取精神和责任担当。对于这些问题，我们将不回避、不懈怠，并以高度负责的态度，采取积极有效的措施，切实加以解决。

　　二、2017年工作安排

　　今年是党的十九大召开之年，也是全面落实市第四次党代会决策部署的重要一年，做好政府各项工作至关重要。今年政府工作的总体要求是：以党的十八大和十八届三中、四中、五中、六中全会精神为指导，深入贯彻习近平总书记系列重要讲话精神和治国理政新理念新思想新战略，特别是对江西工作的重要要求，主动适应经济发展新常态，践行新发展理念，以深化供给侧结构性改革为主线，坚持稳中求进，强化创新驱动，深化改革开放，培育壮大新动能，振兴实体经济，加快脱贫攻坚，确保经济平稳健康发展和社会和谐稳定，奋力迈出“三个走在前列”、实现“两大战略任务”⑤的坚实步伐，以优异成绩迎接党的十九大胜利召开。

　　今年全市发展主要预期目标是：生产总值增长9%，财政总收入增长6%，规模工业增加值增长9.5%；固定资产投资增长15%，社会消费品零售总额增长12.5%，实际利用外资增长9%，外贸出口总额增长1%；城镇和农村居民人均可支配收入分别增长8.6%和10%，居民消费价格指数涨幅控制在3%以内，城镇登记失业率控制在4.5%以内，节能减排完成省下达任务。

　　以上目标贯彻了省市党代会和经济工作会议精神，与全市“十三五”规划目标相衔接，并为供给侧结构性改革、发展动能转换留出空间。工作中，我们将牢牢把握稳中求进的总基调，具体做到“三个坚持”：一是坚持进中求快。始终立足发展不足这一基本市情，着力实现经济总量增长幅度、财政总收入增加幅度、城乡居民增收幅度、贫困群众脱贫步伐快于全国全省平均水平，实现加快发展、加快脱贫。二是坚持进中求新。把握新趋势、新机遇，大力实施创新驱动，既发展新兴产业，又挖掘传统产业新内涵，发展新经济，培育新动能。三是坚持进中求好。积极转变发展方式，促进更高质量、更有效率、更加公平、更可持续的发展，推动人民生活水平、社会保障水平与经济发展水平同步提高，让吉安人民的日子过得更好、更加幸福。

　　围绕上述目标，重点抓好以下八个方面的工作：

　　（一）注重供需双侧发力，推动经济平稳较快增长。坚持以创新供给带动需求扩展，以扩大需求倒逼供给升级，全力稳定经济增长。

　　扩大有效投资。优选投资方向，围绕交通能源、基础设施、产业升级、生态环保、民生保障等领域，安排重点项目400个，预计总投资2400亿元，力争年内完成投资900亿元以上；推进昌吉赣客专、蒙华铁路吉安段、广昌至吉安高速、新干航电枢纽、井冈山航电枢纽等在建项目，抓紧做好吉抚武温、渝长厦、咸宜吉、吉安至新余城际铁路等项目前期工作，加快井冈山机场二期扩建，打造全省一流支线机场。拓宽投资主体，既保持政府投资的合理增长，更发挥民间投资的主力军作用，继续推出一批PPP⑥示范项目，落实产权保护相关法规，保护公私产权，让投资者安心、放心。强化争项争资，用好用足苏区振兴对口支援政策，谋划对接一批重大项目，力争项目进笼子、资金进盘子。

　　挖掘消费潜力。坚持把兴业态与拓消费结合起来，开展“增品种、提品质、创品牌”专项行动，促进供需匹配，释放消费潜力。巩固提升传统消费，落实房地产发展举措，培育住房公共租赁市场，满足居民住房刚性需求和改善性需求，切实稳定住房消费；加快新能源汽车充电桩等基础设施建设，落实小排量汽车、新能源汽车税收优惠政策，扩大汽车消费。培育扩大新兴消费，推进宽带网络普及、提速和降费，加快“宽带乡村”建设，促进电商、网购、快递深度融合，不断拓展信息消费；统筹实施旅游休闲、文体教育、养老养生、绿色时尚等消费促进工程。深化质量兴市战略，强化品牌与标准引领，健全消费者权益保护机制，营造良好的消费环境。

　　振兴实体经济。深化“降成本、优环境”专项行动，在落实政策、解决问题、督查考核上再深入，在对口帮扶、金融帮扶、专家帮扶和产业链帮扶上再精准，充分释放政策红利。加大减税降费力度，开展贷款环节收费专项整治，完善货运信息平台，进一步降低企业融资、物流和交易成本。推进“众创业、个升企、企入规、规转股、扶上市、育龙头、聚集群”，引导企业聚焦主业、强化管理，发扬“工匠精神”，做精产品、做优服务，全面提升实体经济实力。

　　（二）注重新旧动能协同发力，促进产业转型升级。抓住新一轮科技革命的机遇，培育新动能，提升传统动能，促进新旧动能融合发展、产业迈向中高端。

　　强化创新引领。坚持以科技创新为核心，积极对接全省创新驱动“5511”工程⑦，增加科技专项经费，实施一批重大科技专项。突出企业创新主体地位，鼓励企业增加研发投入，创建重点实验室和工程技术中心，突破关键技术，抢占技术制高点。强化产学研协同创新，每个县市区建立一个以上产业技术创新联盟，每个工业园区建成一个生产力促进中心，全市新增高新技术企业20家以上。

　　培育壮大新经济。发展新制造经济，聚焦电子信息首位产业，组建电子信息技术研究院，加快建设软件产业园，创建省级制造业创新中心，实施“制造业50强培育计划”，推动合力泰跻身100亿企业行列，红板过70亿、木林森过50亿，力促TFT⑧项目落户，力争电子信息产业主营业务收入突破800亿元；开展“机器换人”专项行动，促进个性化定制、智能化生产。发展新服务经济，突出发展现代旅游，实施“全景吉安、全域旅游”战略，加快井冈山市、青原区、安福县国家全域旅游示范区建设，启动青原山、羊狮慕国家5A级景区创建；加强与省内外旅游投资集团战略合作，推进旅游资源的集成整合，加快永和吉州窑、泰和槎滩陂、万安花花世界、遂川最美梯田等开发，打造旅游目的地集聚区；加强旅游营销，不断唱响吉安旅游品牌。发展智慧经济，深入推进“互联网 ”行动计划，实施电子商务提升工程，加快电商产业园建设，年内全市所有乡镇和行政村实现电商服务站点全覆盖，争取国家跨境电商试点，促进网络与实体、线上与线下互动发展。

　　改造提升传统产业。推进工业强市战略，以“互联网 ”、跨界融合、分享经济等新思维，促进工业量质齐升，力争全市规模工业增加值达到830亿元。打好企业技术改造、企业管理提升和工业园区完善提升“三大战役”，策应全省企业技改“三千计划”⑨，以新技术、新工艺、新标准，嫁接改造装备制造、食品医药、化工建材等传统产业，向高端化、智能化、绿色化方向发展。

　　加快集聚要素资源。强化金融支持，争取中信银行、兴业银行在吉安设立分支机构，稳步发展第三方支付、P2P网贷⑩、众筹等互联网金融产品，构建覆盖面广、持续性强的普惠金融服务体系，力争金融机构新增贷款260亿元、增长20%以上；抓住IPO扶贫政策輥輯訛机遇，推进企业上市，力争海能实业首发上市、井冈山旅游成功上市，全市新增10家企业在“新三板”挂牌，50家企业在区域性股权交易市场挂牌；落实“三金并举”輥輰訛，支持社会资本参与设立产业基金；推动险资入吉。强化人才支撑，深化“回归家园、奉献家乡”专项行动，发挥驻外机构、商会协会的桥梁作用，吸引吉安籍人才返乡创新创业；开展本土优秀企业家高端培训，培养一批富有战略眼光和创新精神的领军企业家。

　　（三）注重区域城乡统筹联动，加快构建多元支撑格局。坚持区域协同、城乡统筹，优化生产力布局，在协调发展中壮大实力、增强后劲。

　　提升吉泰走廊优势板块。推进产业集聚，加快井冈山经开区金鸡湖新区开发建设，推动医药产业园、中韩产业园、井大科技园、循环产业园建设，加快形成电子信息首位产业和先进制造、生物医药、新能源新材料、绿色食品产业格局，打造全省战略性新兴产业集聚区，力争经开区工业主营业务收入突破700亿元；推进吉安高新区提质增效和基础设施建设，加快体制机制创新，聚焦数字视听等高新产业，打造“中部声谷”，力争主营业务收入突破400亿元；完成井冈山机场临空经济区规划，争取桐坪航空产业园纳入国家通航产业示范区试点，支持吉水县军民融合产业园打造省级军民融合产业基地，促进走廊内各县区差异化联动、融合式发展。完善基础设施，落实吉泰城镇群规划，做好吉安高铁西站至井冈山机场、中心城区至桐坪机场快速通道建设前期工作；推进105国道“东改工程”，完成线形规划，启动吉水、泰和段建设。提升公共服务水平，完成城北水厂主体工程建设，推进螺子山污水处理厂二期扩建，加快走廊供电、供气和供排水一体化。

　　打造高铁经济潜力板块。对接全国全省高铁网络布局，编制吉安高铁经济发展规划。策应昌吉赣客专建设，统筹沿线土地开发、站点建设和城市发展，布局一批旅游、商贸、物流等特色产业。加快中心城区高铁新区建设，年内全面启动高铁站房、周边路网，以及君华大道、阳明西路地下综合管廊等基础设施建设，尽快拉开新区框架。

　　做强县域经济支撑板块。支持各县市区培育1—2个主导产业，提升狗牯脑茶、泰和乌鸡、安福火腿、永新蚕桑、永丰油茶、峡江医药等特色产业，加快万安声学元器件生产基地建设，力争泰和触控显示、新干盐卤药化进入百亿集群行列，井冈山经开区通讯终端主营业务收入突破200亿元。鼓励县市区运用金融和资本手段实施一批交通升级项目。深化安福省直管县改革，落实扩权强县措施，赋予县域更大的自主发展权。

　　夯实城乡发展重点板块。做优中心城区，提升庐陵新区核心带动力，加快赣西堤建设，推进神岗山大桥和吉安大桥西岸互通项目，实施“点亮吉安”和畅通“微循环”工程，年内打通48条“断头路”，完成10个停车场建设，新增停车位3000个以上；引导网约车行业健康发展，优化公共自行车网点布局，并向县域拓展，继续投放一批新能源公交车，全面改造公交候车站亭；加快智慧城市建设，年内实现大数据中心和公共服务平台上线运行，促进市民就诊、医保、公交“一卡通”；推进“海绵城市”輥輱訛试点，发展装配式绿色建筑；强化城市管理，加快构建大部制城管体制，提高社区服务和物业管理水平，做好国家卫生城市复审和第五届全国文明城市参评迎检工作，深化违法搭建、超店经营专项整治，让“清、净、整洁”体现到每个角落。做强县市城区，实施一批文化修复和特色街区建设项目，促进县城提质扩容。做精特色小镇，推进扩权强镇，年内每个县市区打造1个以上产业有特色、形态有特点、文化有特质的特色小镇，建设一批有活力、有实力的中心镇。做美宜居乡村，完成3000个美丽乡村点建设，突出抓好“七改三网”輥輲訛，强化农村建房管控，加强“空心村”整治，加大乱埋乱葬专项治理，加快建设公益性墓地；发展多种经营、股份合作，用活集体产权，壮大村集体经济；加强乡风文明建设，关心农村“留守群体”，共建共享“整洁美丽、和谐宜居”的美丽乡村。

　　（四）切实强化改革攻坚，增强发展动力活力。坚持以供给侧结构性改革为主线，全面推进重点领域和关键环节各项改革，进一步激发市场活力和社会创造力。

　　大力推进“三去一降一补”輥輳訛。运用市场和法治的办法，坚决淘汰落后产能，有序化解煤炭等行业过剩产能。坚持房子的居住属性，支持居民合理自住购房，提高棚改货币化安置比例，控制商品住宅库存，促进房地产行业健康发展。支持企业开展市场化、法治化债转股，降低企业负债水平；强化政府性融资平台负债监测，防范金融债务风险。

　　积极推进农业供给侧结构性改革。着力稳定粮食生产，加强耕地数量、质量、生态“三位一体”保护与管控，加大高标准农田和农业基础设施建设，提高农业综合生产能力。着力优化绿色农产品供给，实施井冈蜜柚“6611”工程輥輴訛，申报注册井冈蜜柚地理标志商标和生态原产地产品保护认证，大力发展有机农业，促进农业规模化、标准化、生态化和品牌化。着力增强农业效益，推进农产品精深加工，发展家庭农场，培训职业农民，整合提升现代农业示范园，争创一批省级重点示范园；抓好农村土地“三权分置”輥輵訛和“两权”抵押贷款輥輶訛改革试点，不断激发农村产权市场的活力，促进农业增效、农民增收。

　　深入推进政府“放管服”综合改革。着力增强放权的协同性，继续精简一批行政审批事项，削减归并一批前置审批事项，全面推行工商登记“五证合一”“一照一码”，完善行政权责动态调整机制，落实权责清单管理，确保真放权、放真权。着力提高监管的有效性，推进“双随机一公开”輥輷訛监管全覆盖，健全守信联合激励、失信联合惩戒机制，让守信者一路畅通、失信者处处受限。着力强化服务的便利性，推行“互联网 ”政务服务，实施企业注册登记网上并联办理，编制发布市县公共服务事项清单，健全乡镇便民服务中心，让群众办事更加便捷。

　　统筹推进各项重点改革。深化财税体制改革，强化政府债务管理，加快项目支出执行，健全税收共治格局。推进国资国企改革，完善法人治理结构，发展混合所有制经济。整合公共资源交易平台，逐步将工程建设、政府采购、国土资源、国有产权等纳入统一交易范围，提高公共资源配置效率。推进事业单位分类改革，稳步启动事业单位公车改革。

　　（五）持续扩大双向开放，打造内陆开放新高地。积极融入“一带一路”、长江经济带、京津冀协同发展国家战略，推进更高水平的全面开放、双向开放，打造开放发展新优势。

　　高水平引进来。扭住招商引资“一号工程”不放松，推动招商引资提质增效。强化精准招商，瞄准长珠闽、京津冀、港澳台等重点区域，坚持行业领衔、市县互动、县县联动，开展“点对点”专项招商，增强招商实效。强化招大引强，实施外企、央企、台商、吉商入吉行动，健全重大项目成果共享和激励机制，加强与世界500强企业国内办事处的联络，努力在引进行业龙头上取得新突破。强化方式创新，推进股权招商、PPP项目招商和IPO扶贫招商，力争招引一批总部企业落户吉安。

　　大力度走出去。实施外贸“优进优出”行动计划，设立外贸周转金，实行出口退税企业质押抵押贷款。引导生产企业出口全申报，提高电子信息、先进制造、特色食品和农产品出口比重，促进井冈蜜柚出口。设立“走出去”引导基金，组织开展“百家企业出境参展”活动，支持优质企业融入全球产业链。

　　全方位增优势。优化功能性平台，加快外贸综合服务中心建设，完善井冈山出口加工区功能，开通吉安至深圳盐田港铁海联运快速班列，力争省进出口机械电子产品安检实验室整体搬迁；推进通关通检便利化，建立国际贸易“单一窗口”輦輮訛新型通关模式。完善园区服务配套，建成标准厂房100万平方米、保障性住房3000套以上。进一步优化投资环境，营造“亲清”政商关系，打造开明、诚信、清廉的环境品牌。

　　（六）加强生态保护与建设，厚植绿色发展优势。牢固树立“绿水青山就是金山银山”的理念，着力治山理水、显山露水，努力在打造美丽中国“江西样板”上走在前列，让蓝天常驻、青山常在、绿水长流。

　　强化生态环境治理。坚持源头严防，严把环评准入关，对环保可信度不高的项目，坚决不引进、不实施。坚持过程严管，坚决抓好中央环保督察组反馈问题的整改，持续向环境污染“开战”；加强空气质量自动监测，狠抓施工扬尘、工业废气、机动车尾气污染治理，确保空气质量保持在国家二级标准以上；严格落实水资源管理“三条红线”輦輯訛，开展赣江吉安段环保专项整治，巩固“清河行动”成效；统筹布局全市垃圾处理项目，启动中心城区垃圾焚烧发电项目，完成樟山垃圾填埋场升级改造，年内每个县市区新增2个以上乡镇开展垃圾处理标准化试点，统一推行“户分类、村收集、镇转运、县处理”模式，促进垃圾处理减量化、资源化、无害化；加强农村污水处理，推动配套管网向中心村延伸，推行农村污水“生态疗法”輦輰訛。坚持后果严惩，推进环保监察监测机构垂直化管理，加强环保执法，以“零容忍”态度惩处环境违法行为。

　　提升生态建养水平。着力造绿添绿，推进森林质量提升工程，实施新一轮退耕还林、山水林田湖生态修复等生态工程，年内完成人工造林25万亩、森林抚育100万亩；规划建设一批原生态的小游园和公共绿地，力争全市人均公园绿地面积达到16.5平方米；加快井冈山、遂川、万安、安福、永新国家重点生态功能区建设。着力倡绿护绿，实施“生态 ”行动，推进国家低碳城市、国家循环经济示范城市试点，稳步发展风电光伏清洁能源，培育节能环保产业，不断提高经济绿色化程度。

　　健全生态长效机制。探索生态补偿、水权交易市场化机制，争取流域生态补偿资金支持。落实生态环保责任，以深化赣江吉安段“河长制”为重点，推动“河长制”延伸到每一个行政村，落实到每条河渠、每口山塘；严格实行生态环境损害终身追责制，推行领导干部生态环境保护与自然资源资产离任审计。健全生态文明宣传教育机制，弘扬生态文化，倡导低碳生活。

　　（七）织牢民生保障网，不断增强人民群众获得感。始终坚持民生为本、民生为重，加强普惠性、基础性、兜底性民生建设，集中办好一批民生实事，不断抒写温暖幸福的民生答卷。

　　坚决打好精准脱贫攻坚战。严格落实脱贫攻坚“军令状”，巩固井冈山市、吉安县脱贫成果，总结推广经验，加大工作力度，确保年内全市农村贫困人口再减少5万人以上，贫困村退出200个以上，万安县、永新县实现脱贫摘帽。突出产业脱贫，推行“四个一”产业扶贫輦輱訛，实施旅游扶贫、电商扶贫。突出健康脱贫，筑牢基本医保、大病保险、商业保险、医疗救助“四道防线”，年内为全市所有建档立卡农村贫困人口办理重大疾病商业保险。统筹推进保障脱贫，实现农村危旧土坯房“应改尽改”，完成移民搬迁1万人。注重精神脱贫，坚持“志智双扶”，激发贫困群众脱贫内生动力，决不“养懒汉”。严格评估脱贫成效，严防严查“数字脱贫”和“虚假脱贫”，努力实现更高质量、更可持续的脱贫。

　　着力促进创业就业。打造一批省级创业孵化基地和创客空间，以创业带动就业。落实国家创业担保贷款新政策，新增发放创业担保贷款10亿元以上。做好高校毕业生、农民工、困难群众等重点群体就业工作，力争全市新增城镇就业4.5万人、转移农村劳动力6万人。

　　完善社会保障体系。推动城乡医疗保险和养老保险“全覆盖”，落实失地农民养老保险措施。加快城乡居民医保并轨运行，推进异地就医即时结算，探索县域内困难群众住院“先诊疗、后付费”机制。稳步提高低保水平，加强农村低保与扶贫脱贫的衔接。兴建一批县级福利院，积极发展民办养老机构，健全街道社区养老机构，探索居家养老、社区养老新模式。加强优抚安置工作，推进军地融合发展。新开工改造各类棚户区4520户，基本建成公租房5096套，实现住房困难群众“出棚进楼”安居梦。

　　（八）推动社会事业全面发展，促进社会和谐稳定。紧紧围绕人民群众需求，加快补齐社会事业短板，推动社会全面进步。

　　优先发展教育事业。优化学前教育布局，扩大普惠性幼儿园覆盖面，加大乡镇公办幼儿园建设力度，年内实现乡镇中心幼儿园全覆盖。推进义务教育公办学校标准化、信息化建设，确保县市区义务教育均衡发展国家评估通过率达到90%以上。扩大城区办学规模，年内新增学位5万个以上，有效化解城区“大班额”问题。逐步推进高中义务教育。加快职业教育发展，打通中职到高职的直升通道，加快职教园区建设，促进产教融合。支持井冈山大学创办厦坪校区。

　　繁荣发展文化事业。实施文化惠民工程，推动精品文化下乡村、进社区，年内20%的村居和社区建成文化中心。实施全民阅读促进工程，加强数字群艺馆、图书馆、档案馆、方志馆建设，推进文化场馆免费开放，促进文化资源共用共享。实施“文化 ”工程，加强庐陵文化研究开发，打造庐陵人文谷等文化产业基地，创建国家级庐陵文化生态保护试验区。隆重纪念井冈山革命根据地创建90周年，提升井冈山景区建设和服务水平，加强红色资源开发利用，推出红色精品，讲好红色故事，推动弘扬井冈山精神走在前列。

　　推进健康吉安建设。启动城市公立医院改革，推进医疗、医保、医药“三医”联动，提升基层医疗服务能力，构建分级诊疗体系，改善群众就医体验。加强医疗机构建设，确保市儿童医院建成运营、市中心人民医院内儿科医技大楼建成投用，加快建设市人口健康大数据平台和区域医学影像中心。培育大健康产业，发展中医药事业，鼓励社会资本兴办护理康复、养老养生等“医养结合”新型机构，建设生态养生之都。做好新形势下计划生育工作，加强妇幼保健和优生优育工作。健全食品药品质量监管和保障体系，治理“餐桌污染”，让人民群众吃得放心、用得安全。完善体育健身基础设施，稳步推进学校和机关单位体育场馆免费向社会开放，办好吉安市第五届运动会，以全民健身促进全民健康。

　　切实加强社会治理。推进法治吉安建设，规范信访秩序，加大依法治“闹”力度，进一步营造信法、用法的浓厚氛围。深化平安吉安建设，提高打击新型犯罪、经济犯罪和多发性违法犯罪的能力，健全安全风险滚动排查、监测预警、应急处置机制，推进公共安全视频监控联网应用工程，完善立体化、信息化治安防控体系。严格落实安全生产责任制，坚决遏制重特大事故发生，加强自然灾害等突发事件预警信息发布工作，切实保障人民群众生命财产安全。

　　各位代表，今年各项目标任务，对政府工作提出了新的更高要求。我们将持续加强政府自身建设，不断提高工作水平。始终恪守为民之责，坚持“以百姓之心为心”，时刻谨记公仆身份，沉到一线、下到基层，与老百姓同坐一条板凳，做到重大决策为民而定、政策举措应民而出、安危冷暖唯民所系，用沾满泥土的双脚，去丈量我们每一名干部为民服务的真情。自觉秉持法治之器，坚持政府常务会学法制度，强化决策程序的法定约束，推动政府行政管理立法；自觉接受人大及其常委会的法律监督和政协的民主监督，主动接受司法监督和舆论监督，认真办理人大代表建议和政协提案，广泛听取民主党派、工商联、无党派人士和各人民团体的意见。切实践行实干之风，坚持在继承中创新，一任接着一任干，扑下身子抓落实；完善重大项目领导挂点、重点工作现场办公、重要事项督促检查机制，严查懒政、怠政行为，切实克服不作为、慢作为、乱作为现象；健全容错纠错机制，为负责者负责，为担当者担当，不断形成“干事扛事成事”的鲜明导向。着力传扬清廉之德，坚决贯彻中央八项规定精神以及省市有关规定，严肃查处发生在基层和群众身边的腐败行为；严格控制“三公”经费，把有限的财政资金用在刀刃上；加强对公职人员的教育、管理和监督，努力打造一支政治坚定、人民满意的政府公务员队伍。

　　各位代表，“草木蔓发，春山可望”。吉安加快发展其时已至、其势已成，让我们更加紧密地团结在以习近平同志为核心的党中央周围，在市委的坚强领导下，大力弘扬跨越时空的井冈山精神，解放思想、改革创新，团结一心、奋勇拼搏，以优异成绩迎接党的十九大胜利召开，为推动吉安实现“三个走在前列”“两大战略任务”而努力奋斗！
